# Development of a Screening Intervention for Dysphagia in Hospitalised Geriatric Patients

**DOI:** 10.1007/s00455-025-10803-9

**Published:** 2025-01-30

**Authors:** Anne Mette Schmidt, Helene Nørgaard Kristensen, Dorte Melgaard, Asger Roer Pedersen, Lene Mark, Charlotte Weiling Appel, Sofie Langergaard, Charlotte Overgaard

**Affiliations:** 1 Medical Diagnostic Centre, University Clinic for Innovative Patient Pathways, Regional Hospital Central Jutland, Silkeborg, Denmark; 2 Department of Acute Medicine and Trauma Care, Aalborg University Hospital, Aalborg, Denmark; 3 Department of Clinical Medicine, Aalborg University, Aalborg, Denmark; 4 Department of Public Health and Epidemiology, The Faculty of Medicine, Aalborg University, Aalborg, Denmark; 5 The Unit of Health Promotion, Department of Public Health, University of Southern Denmark, Esbjerg, Denmark

**Keywords:** Deglutition disorders, Mass screening, Early medical intervention, Aged, Geriatric assessment, Inpatients

## Abstract

Prevalence of dysphagia is high in hospitalised geriatric patients, posing risks of complications including malnutrition, dehydration, aspiration, and pneumonia. These complications may lead to reduced daily functioning, frailty, prolonged hospital stays, readmissions, and mortality. Diagnosing dysphagia in geriatric patients is often challenging due to the complex health conditions of this patient group, and overall these patients are at risk of lack of continuity in patient pathways and unnecessary hospitalisations. Recognising the critical importance of prompt diagnosis and treatment of dysphagia, we developed a dysphagia screening intervention aligned with clinical guidelines and the political focus to improve patient pathways and reduce preventable hospitalisations. This article outlines the development process of a dysphagia screening intervention to geriatric patients (≥ 65 years) admitted to medical inpatient wards. We applied a theory-, evidence- and implementation-based approach combined with stakeholder involvement in adherence to the IdentifyiNg and assessing different approaches to DEveloping compleX intervention (INDEX) guidance, encompassing eleven actions. We developed a dysphagia screening intervention comprising a screening procedure (the 4 Questionnaire Test (4QT), the 30 ml water swallowing test, and an action algorithm) targeting the patient level. Moreover, we developed an implementation strategy (activities necessary for adequate delivery of the dysphagia screening procedure and activities supporting the delivery of the screening procedure) targeting health professionals and the organisational level. The dysphagia screening intervention is now ready for feasibility testing, promising improved health and healthcare services for hospitalised geriatric patients.

## Introduction

Swallowing disorders, or dysphagia, is a common geriatric syndrome like dementia, falls, and osteoporosis [1]. Dysphagia affects between 23 and 82% of hospitalised patients [2] and prevalence increases with age. Alterations in the aero digestive tract due to aging reduce swallowing efficiency and safety, but comorbidity and increased use of medications can also cause dysphagia [3, 4]. The consequences of dysphagia lead to various complications, including aspiration, pneumonia, malnutrition, and dehydration [4–6]. Over time, these complications may lead to a reduction in activities of daily living, frailty, lower quality of life, social withdrawal, increased length of hospital stay, readmission and mortality [1, 5, 7–9].

The geriatric patient is often complex and presents with multiple illnesses, polypharmacy, as well as physical, mental and cognitive challenges [10]. The diagnostic picture of the geriatric patient may thus be blurred [11] and patients have an increased risk of experiencing lack of coherent patient pathway as well as unnecessary admissions and readmissions [10]. Dysphagia thus adds to the complexity of managing geriatric patients [12] and early diagnosis is necessary to initiate timely treatment, and thereby avoid consequences and complications [11, 13]. International white papers by The European Society for Swallowing Disorders and a Danish guideline recommend systematic screening for dysphagia in geriatric patients [1, 14, 15]. Although multiple screening tools are available, there is no consensus on which tools and/or procedures to apply in screening for dysphagia in geriatric patients [16].

Thus, there is a need for a systematic screening for dysphagia in geriatric patients in clinical practice, ensuring coherent patient pathways and reducing preventable hospitalisations [17].

We have developed a dysphagia screening intervention to optimise patient pathways for hospitalised geriatric patients, and improve health and healthcare service for these patients. This study describes the development of the dysphagia screening intervention comprising a dysphagia screening procedure targeting geriatric patients, and an implementation strategy targeting health professionals and the organisational level.

## Methods

### Design

To ensure rigorous conduct and reporting of the development process, we used the IdentifyiNg and assessing different approaches to DEveloping compleX intervention (INDEX) guidance consisting of eleven actions [18] (Table 1). To improve understanding of the development process and readability of the present paper, the eleven actions will be reported sequentially in the Result section. To further improve the quality of reporting, the guidance for reporting intervention development studies in health research was used as a checklist [19]. This research project was overall guided by the British Medical Research Council framework for developing and evaluating complex interventions [20].

**Table 1:** The eleven actions applied [18] in the development of a dysphagia screening intervention

Number	Action
1	Plan the development process: (i) Understand the problem, (ii) Identify resources– time and funding, and (iii) Decide which approach to intervention development to take
2	Involve stakeholders, including those who will deliver, use and benefit from the intervention
3	Bring together a team and establish decision-making processes
4	Review published research evidence
5	Draw on existing theories
6	Articulate programme theory
7	Undertake primary data collection: (i) Identification of local screening procedures, (ii) Identification of national screening procedures, and (iii) Group interviews with health professionals
8	Understand context
9	Pay attention to future implementation of the intervention in the real world
10	Design and refine the intervention
11	End the development phase

### Setting and Target Population

The dysphagia screening intervention was developed for geriatric patients (≥ 65 years) admitted to medical inpatient wards at Danish hospitals.

In Denmark, five geographical and administrative Regions are responsible for delivering hospital healthcare services. Healthcare in Denmark is primarily tax financed and there is free and equal access for all Danish citizens.

### Stakeholders

We conducted a stakeholder analysis to identify our stakeholders, assess their interest in and influence on the screening intervention, evaluate how the intervention would impact them, and grouped them into five groups according to these insights: (1) health professionals, (2) electronic health record specialists, (3) dysphagia specialists (in Denmark this is occupational therapists), (4) specialists in teaching methods, and (5) management representatives. All stakeholders identified agreed to participate. Further stakeholder details are provided in the Results section.

### Primary Data Collection

#### (i) Identification of Local Screening Procedures

We identified local dysphagia screening procedures at medical inpatient wards and outpatient clinics at regional hospital 1 and 2 on Central Denmark Region electronic platform for detailed descriptions of procedures.

#### (ii) Identification of National Screening Procedures

Denmark is divided into five administrative regions, each responsible for managing and administering secondary healthcare in its area. Each region has between 7 and 13 hospitals. From October to December 2022, we contacted the physiotherapy and occupational therapy departments at every hospital in Denmark, as occupational therapists are the dysphagia specialists in the country. They provided us with information on dysphagia screening procedures in the inpatient wards and outpatient clinics they were affiliated with, in total 126.

A semi-structured interview guide inspired by the Template for Intervention Description and Replication (TIDieR) checklist [21] was used to ensure comprehensive data collection on existing screening tools and screening procedures, as well as health professionals’ experiences using these. Each telephone interview lasted 10–15 min.

#### (iii) Group Interviews with Health Professionals

In November 2022, we conducted two group interviews among a purposive sample of seven health professionals from four medical inpatient wards using a semi-structured interview guide (Table 2). Written consent was obtained prior to the interviews, which were recorded and transcribed verbatim.

**Table 2:** Description of the two group interviews with health professionals from medical inpatient wards

Date in 2022	Duration (minutes)	Setting	Informants	Themes and ideas addressed in the two group interviews
Nov. 8th	48	Two medical inpatient wards, Regional hospital 1, Central Denmark Region	One nurse One occupational therapist specialised in dysphagia	Description of existing dysphagia screening tool(s) and procedure(s), and existing knowledge and skills to identify patients with dysphagia. Description of health professionals taking part in existing dysphagia screening procedure. Identification of barriers and facilitators to implement a dysphagia screening procedure. Presentation and discussion of preliminary ideas for a dysphagia screening intervention.
Nov. 17th	46	Two medical inpatient wards, Regional hospital 2, Central Denmark Region	Two nurses Two social and healthcare assistants One occupational therapist specialised in dysphagia	

### Primary Data Analysis

#### (i) Identification of Local Screening Procedures

In the first step of the primary data collection, identified documents on local screening procedures were entered into tables inspired by the TIDieR checklist [21] to facilitate easy comparison of the identified screening procedures. Health professionals confirmed agreement with existing clinical practice in the validation of the identified screening procedures.

#### (ii) Identification of National Screening Procedures

Tables inspired by the TIDieR checklist [21] were completed for each of the 126 medical inpatient wards and outpatient clinics in Denmark with a short description of their clinical practice. Wards performing dysphagia screening procedures were described in more detail.

#### (iii) Group Interviews with Health Professionals

We used thematic analysis to identify, analyse, organise, describe and report identified themes [22]. Thematic analysis is useful to examine different perspectives and to highlight differences and similarities, as well as generate unanticipated insights [23]. We familiarised ourselves with the data by reading notes and transcriptions. Then initial codes were developed and discussed deductively matching the interview questions and grouped into common themes. Subthemes were formed inductively. Researcher triangulation was used in all steps of the analysis.

## Results

The dysphagia screening intervention was developed from January 2022 to October 2023. The results of the iterative development process are reported according to the eleven actions for intervention development [18].

### Action 1 Plan the Process

#### (i) Understand the Problem

Two occupational therapists specialised in dysphagia mentioned the lack of an applicable and useful systematic screening procedure. They requested a simple and relatively easy screening procedure that both followed clinical guidelines on dysphagia and met the overall political focus on ensuring coherent patient pathways to reduce preventable hospitalisations in geriatric patients.

#### (ii) Identify Resources– Time and Funding

A local Clinic, Education and Research Community about Rehabilitation provided financial support to apply resources to complete a thorough problem identification, draft a scientific protocol, and to initiate application for funding.

#### (iii) Decide Which Approach to Intervention Development to Take

O’Cathain et al. have described several approaches to intervention development [18, 24]. We combined approaches and applied an evidence- and theory-based as well as an implementation-based approach to fit our specific problem, intervention, and setting [18, 24].

### Action 2 Involve Stakeholders

The development team identified and involved a range of stakeholder groups with different areas of expertise and different backgrounds. Thus, the stakeholders were involved when their knowledge and skills were needed. We met with the different stakeholder groups ad hoc either face-to-face or online and corresponded by email. Some stakeholder groups were only involved a few times while others were involved daily for a period. Collectively, the combined knowledge and competences of all the stakeholders qualified the development team to make the final decisions (Table 3).

**Table 3:** Stakeholder groups, participants, and result of involvement

Stakeholder group	Participants	Result of involvement
Health professionals	Three nursing staff (one responsible for nutrition), and three occupational therapists (responsible for dysphagia management) from Regional hospital 2, Central Denmark Region.	They gave insight into the target population, the setting, and their needs and priorities, and generated ideas for the dysphagia screening intervention. Further, they addressed potential barriers e.g., patients who cannot complete the screening procedure due to delirium or dementia. The health professionals stressed that documentation in the electronic health record should be user-friendly.
Electronic health record specialists	A physiotherapist from Regional hospital 2, Central Denmark Region. A health IT consultant, Central Denmark Region.	They provided knowledge about both the back-end and front-end of the electronic health record, which ensured that documentation was user-friendly and intuitive, and that relevant data could be extracted for the purpose of research.
Dysphagia specialists	An occupational therapist from Regional hospital 2, Central Denmark Region. A physiotherapist and a nurse from a Regional hospital, Region Zealand.	Perspectives from this multidisciplinary group from different clinical settings ensured that the dysphagia screening procedure was simple and relatively easy to deliver in daily clinical practice, and transferable to other target populations and settings.
Specialists in teaching methods	An occupational therapist from VIA University College (university of applied sciences). Two occupational therapists from Regional hospital 2, Central Denmark Region. An e-learning consultant, Central Denmark Region.	These specialists were involved in the development and conduct of teaching and training sessions and e-learning activities. This enhanced the quality and ensured that the delivery of the dysphagia screening procedures was clinically relevant to the target population and settings.
Management representatives	A Clinical Director (registered nurse), a Clinical Lead (registered nurse), a Senior Consultant in geriatrics, and a Clinical Lead (Occupational therapists/Physiotherapists) from Regional hospital 2, Central Denmark Region. A Clinical Director (registered nurse), and a Clinical Lead (Occupational therapists/Physiotherapists) from Regional hospital 1, Central Denmark Region.	They endorsed the dysphagia screening intervention as a vital component in the patient pathway, providing the necessary support and resources for completing the dysphagia screening procedure.

### Action 3 Bring Together a Team

As described in Action 1, this research project started in a local Clinic, Education and Research Community about Rehabilitation where a development team was formed between author AA (qualitative researcher with knowledge about dysphagia, the target population, and clinical practice), and author BB (quantitative researcher with knowledge and experience with research within complex interventions and patient pathways). Author CC (researcher, specialist in dysphagia, and chairwoman of Danish Society and Swallowing Disorders), author DD (qualitative researcher and intervention design specialist with a strong track record in research within complex interventions in various target populations and settings), and author EE (statistician with exhaustive knowledge of and skills in use of data from the electronic health record in patient pathway research), were invited to join the team. The development team made the final data-based decisions during the development process.

### Action 4 Review Published Evidence

A systematic search was conducted in PubMed to identify existing evidence related to physiology, anatomy, prevalence, complications, consequences, recommendations, screening tools, procedures, and interventions in dysphagia. The review of evidence was an iterative process conducted throughout the development process. We identified a review estimating the prevalence of dysphagia in hospitalised older adults [2], and several studies describing short term complications [4–6] and long term consequences of dysphagia [1, 5, 7–9] for patients and the health care system. These studies provided knowledge about the epidemiological context.

We also identified two international white papers by The European Society for Swallowing Disorders [1, 15] and a Danish guideline [14] advocating for systematic dysphagia screening in geriatric patients. The guidelines stressed the importance of a quick, simple, cost-effective, low-risk, and practical screening tool, but did not recommend any specific screening tool and/or procedure. A literature review identified 27 different dysphagia screening tools in various target populations. Thus, there is currently no widely accepted screening tool in dysphagia [16]. A scoping review aiming to identify and map the psychometric properties of screening tools in older people is ongoing [25], but results have not been published yet.

Based on the existing evidence and the dysphagia stakeholders awareness of ensuring performance of a simple and relatively easy screening procedure, we decided on a screening procedure consisting of three activities:

1) The 4 Questionnaire Test (4QT) consisting of four questions capturing the patient perspective. A validation of the 4QT in 48 frail older patients found a 100% sensitivity and 80.4% specificity when compared to the clinical gold standard of a speech and language therapy assessment, and a positive predictive value of 50% and a negative predictive value of 100% [26]. The 4QT has been translated into Danish and validated in a geriatric population, but results have not been published yet.

2) 30 ml water swallowing test, which is an momentary objective examination to detect aspiration with high accuracy [27].

3) An action algorithm (if earlier diagnosed with dysphagia or if the screening procedure indicates dysphagia) with referral for further assessment and treatment by dysphagia specialists (e.g. occupational therapist, speech-language pathologists), and in the meantime nutritional management including considerations of nil per os (intravenous fluids/nasogastric tube).

When reviewing the published literature, we were aware of any existing dysphagia screening interventions that could be adapted to our setting and target population as recommended [28]. We identified a study introducing a dysphagia screening procedure combining several screening tools to estimate the prevalence of dysphagia in geriatric patients admitted to an acute medical unit [29]. The screening procedure was not systematically developed with involvement of stakeholders, the duration of the screening procedure was unknown, and an implementation strategy was not provided as the screening procedure was delivered by the researchers [29]. In conclusion, the development team decided that development of a new dysphagia screening intervention was required.

### Action 5 Draw on Existing Theories

The Continuity of Care [30] principle supported the theory of change underlying the screening intervention and offered a rationale for our logic model. The Continuity of Care principle was theorised by Haggerty et al. in 2003, and illustrates how individual patients experience integration and coordination of services over a shorter or longer time frame [30]. Later, a model for continuity of care has been introduced [31]. The Continuity of Care principle consist of three dimensions providing different complementary strategies to achieve continuity of care. The inclusion of all three dimensions is believed to increase the likelihood of coherent care and impact care plan, patient safety, risk of readmission, and patient satisfaction [31].

The first dimension is *informational continuity*, which is focused on ensuring that information in relation to disease and the individual person is passed from one health professional to another and from one healthcare event to another. In the dysphagia screening intervention, informational continuity is attempted to be achieved as information about dysphagia is shared among health professionals and settings e.g., by documentation in the electronic health record and in discharge documents.

The second dimension is *management continuity*, which is achieved when care is delivered in a complementary and timely manner. This is especially important in patients with chronic or complex diseases where several providers are involved. In the dysphagia screening intervention, management continuity is pursued by completing the multidisciplinary screening procedure within the first 24 h after admission, preferably before fluids and meals are offered.

The third dimension is *relational continuity*, which is the ongoing relations between a patient and one or more health professionals. This type of continuity bridges past and current care and establishes a link to future care. In practice, establishing a relation between the patient and health professionals at a medical inpatient ward is challenging. Thus, in the screening intervention, relational continuity is attempted by a patient-centred approach considering individual patient needs and resources, and when determining the future plan collectively between the patient and the health professional.

### Action 6 Articulate Programme Theory

**Fig. 1:**
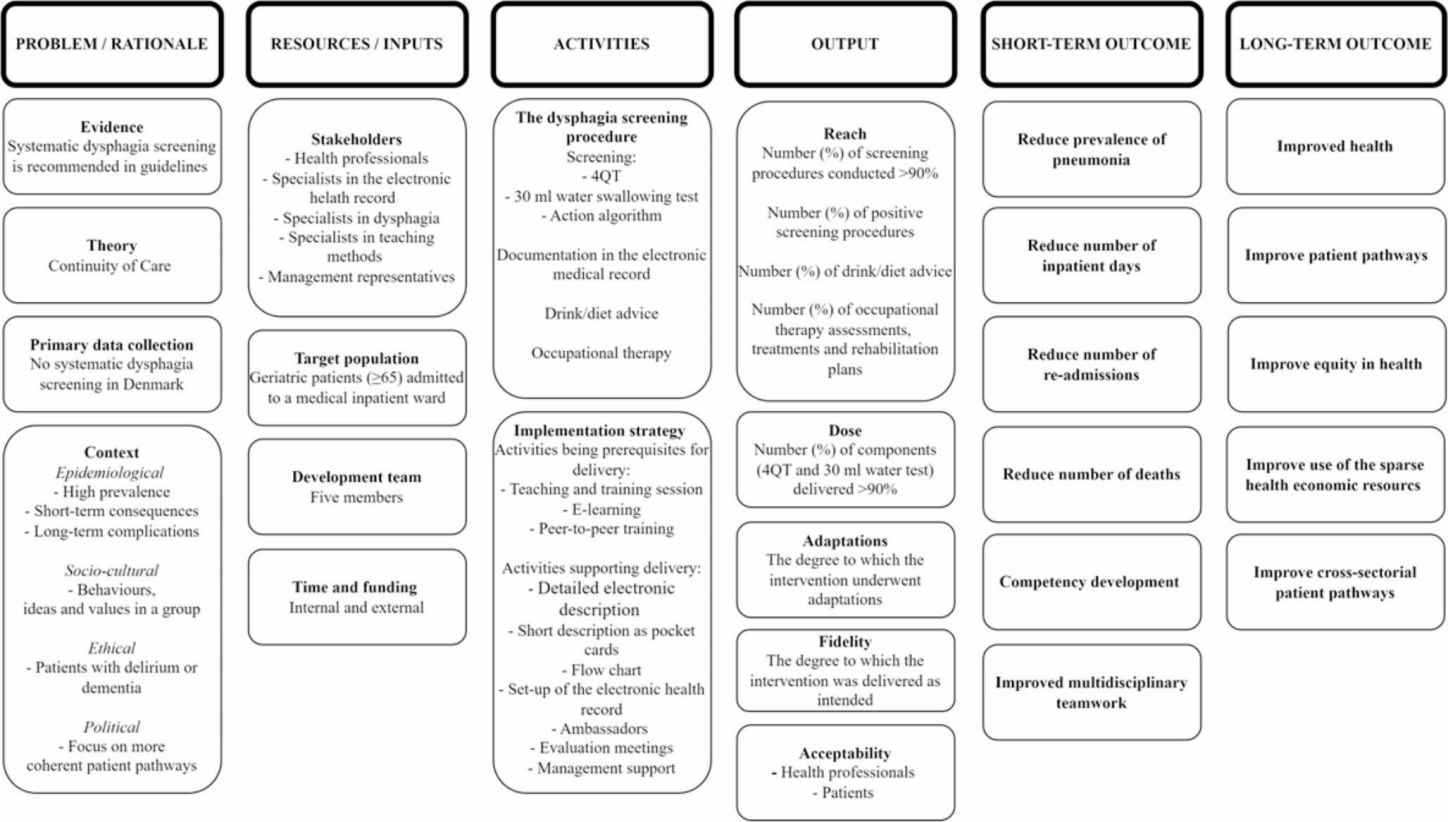
Q1

A logic model is a simple visualisation of the relationship between the rationale behind the screening procedure, the resources and inputs needed, the activities planned, and the expected short and long term results [32]. Our logic model was developed by the development team in an ongoing process, informed by stakeholders, evidence, the Continuity of Care principle, primary data collection, and the understanding of context (Fig. 1). The logic model will be continuously refined during and after a feasibility test, and the underlying assumptions of the screening intervention’s theory of change will be tested alongside an effect evaluation [33].

### Action 7 Undertake Primary Data Collection

The three steps in the primary data collection and analysis were described in the Method section; results are described below.

#### (i) Identification of Local Screening Procedures

Three documents from five medical inpatient wards and one outpatient clinic were identified. Only an acute neurology ward systematically screened for dysphagia using the indirect part of the Gugging Swallowing Screen (GUSS) tool [34].

#### (ii) Identification of National Screening Procedures

In total, nine out of the 126 (7%) medical inpatient wards systematically screened patients for dysphagia (Table 4). We did not identify any ongoing research projects in this field in any Danish hospital.

**Table 4:** Overview of settings, populations and screening tools in systematic screening for dysphagia in danish hospitals

Hospital	Department	Population	Screening tools
University hospital	Geriatric medical inpatient ward	Geriatric	Indirect part of the GUSS^*^
Regional hospital	Geriatric medical inpatient ward	Geriatric	Indirect part of the GUSS
Regional hospital	Orthopaedic inpatient ward	Geriatric	Indirect part of the GUSS
Regional hospital	Medical inpatient ward	Geriatric	GUSS + a questionnaire designed at the department
Regional hospital	Acute inpatient ward	Acute	GUSS
University hospital	Medical inpatient ward	Geriatric	GUSS
Regional hospital	Medical inpatient ward	Geriatric	GUSS + a questionnaire designed at the department
University hospital	Medical inpatient ward	Geriatric	A water swallowing test designed at the department
University hospital	Medical inpatient ward	Geriatric	GUSS

^*^GUSS: the Gugging Swallowing Screen [34]

#### (iii) Group Interviews with Health Professionals

The group interviews revealed that health professionals from the four medical inpatient wards addressed similar topics and concerns e.g., who should be screened, who should deliver the screening procedure, and when to perform the screening procedure. Further, the health professionals provided suggestions and ideas for the screening intervention, including advice to keep it simple and a standard part of clinical practice. A core element identified was that documentation in the electronic health record should be user-friendly, and that information on dysphagia status had to be easily accessible.

Nurse 1: “It has to be something (in the electronic health record) that can be ticked, otherwise I think it takes longer to implement (the screening procedure)”.

*(Medical Inpatient ward, Regional Hospital 1)*

Nurse 2: “I would prefer a feature in the electronic health record where you quickly can see if the patient has presented before with dysphagia”.

*(Medical Inpatient ward, Regional Hospital 2)*

Another core element was the importance of accessible to people with specialised knowledge about dysphagia and the screening procedure, as well as continuous introduction to the screening procedure to new colleagues and students.

Interviewer: “What will it take to make it a success?”

Nurse 2: “We need a dysphagia ambassador in the medical inpatient ward”.

Nurse 1: “And key persons”.

*(Medical Inpatient ward, Regional Hospital 3)*

### Action 8 Understand Context

Stakeholder involvement, evidence, and primary data collection enabled understanding of the context. In addition, the Context and Implementation of Complex Interventions framework [35] guided discussion of different context domains. Based on this framework, we identified the geographical, epidemiological, socio-cultural, ethical, and political context domains as the most pertinent to the screening intervention.

When exploring the geographical context, we gained insight into the organisation and different procedures in the existing patient pathways in medical inpatient wards at regional hospital 1 and 2, Central Denmark Region.

Based on evidence, we uncovered the epidemiological context, and found a high prevalence of dysphagia in hospitalised geriatric patients [2], short term complications [4–6], and long term consequences [1, 5, 7–9].

We explored the socio-cultural context comprising behaviours, ideas and values when involving stakeholders and conducting interviews with health professionals. As an example, an occupational therapist specialised in dysphagia highlighted the lack of multidisciplinary communication as a barrier in the management of dysphagia:

“To ensure good multidisciplinary collaboration, it is important that we talk to each other about patients with dysphagia. We are sometimes frustrated if we have made a dysphagia assessment and made restrictions on what the patient can eat and drink. When you then come in on Monday and a steak has been served, it can be frustrating to see that our assessment is not known. We should talk together about this.”

*(Occupational Therapist, Medical Inpatient ward, Regional Hospital 1)*

The interviews with the health professionals revealed that the screening procedure could be challenging in an ethical context. The health professionals addressed concerns about e.g., how to ensure patient autonomy when screening patients with delirium or dementia, who are not cognitively capable of understanding the screening procedure:

“It can be difficult when the patient can’t communicate. Most patients can tell about it (dysphagia); many of our geriatric patients can’t.”

(*Nurse, Medical inpatient ward, Regional hospital 2)*

Finally, the political context was considered highly important to the screening intervention as it comprises prioritisation of and accessibility to health care services, as well as embraces the political focus on more coherent patient pathways and reduction of preventable hospitalisations in geriatric patients [17, 31]. The need for coherent patient pathways is also supported by the Continuity of Care principle.

In all, understanding the context helped to design and refine the screening intervention.

### Action 9 Attend to Future Implementation

It was considered paramount that the developed screening intervention could be implemented and sustained in real world clinical practice. Therefore, implementation was a recurring theme during the entire development process, and the reason for developing an implementation strategy.

### Action 10 Design and Refine

The dysphagia screening intervention comprises a screening procedure targeting the patient level, and an implementation strategy targeting health professionals and the organisational level, described in detail inspired by the Template for Intervention Description and Replication (TIDieR) checklist [21] (Table 5).

**Fig. 2:**
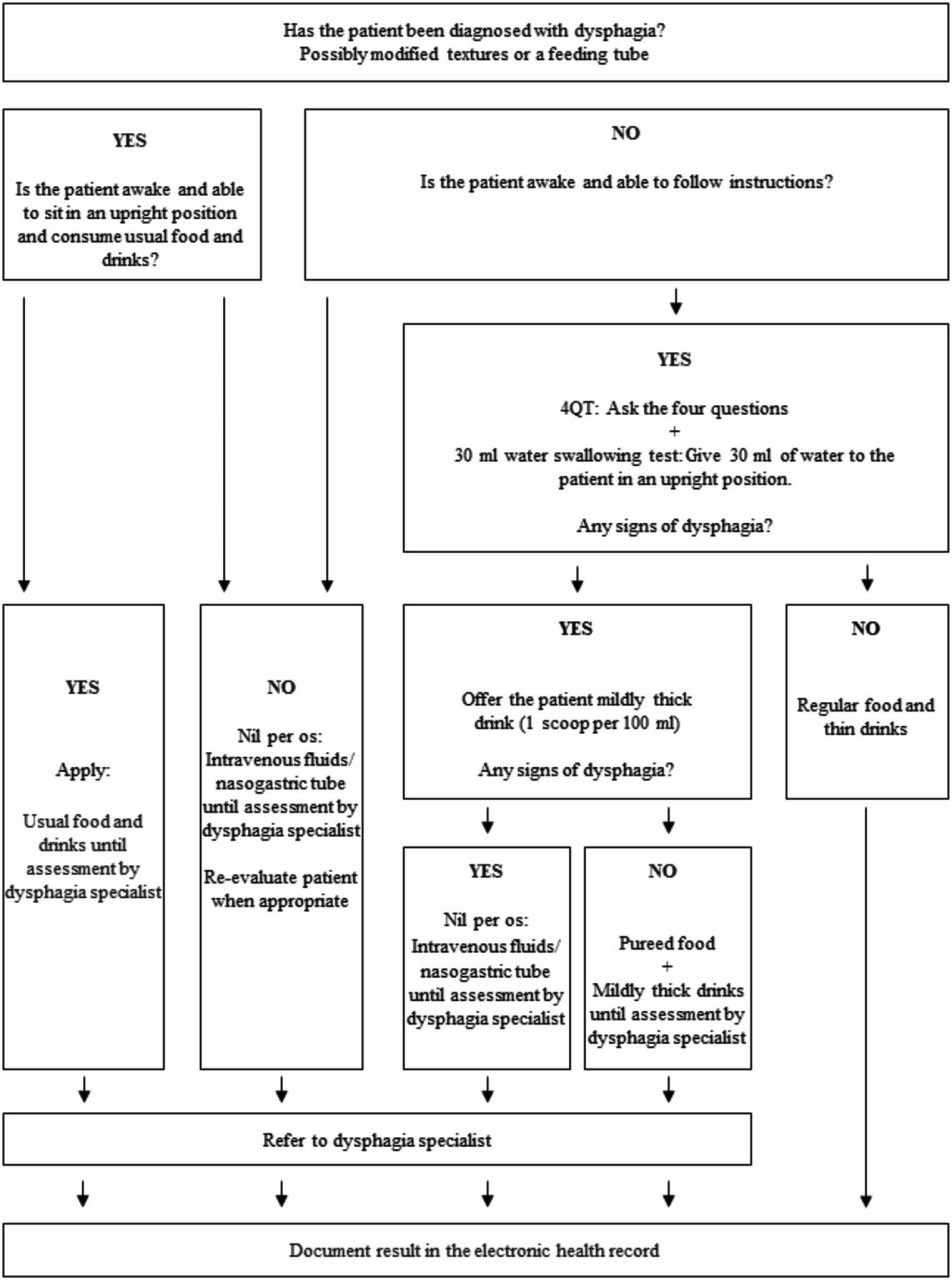
Flow describing the dysphagia screening procedure

The dysphagia screening procedure will be delivered to geriatric patients admitted to a medical inpatient ward by nursing staff, occupational therapists or physiotherapists. It consists of three activities: (1) 4QT [26], (2) 30 ml water swallowing test [27], and (3) an action algorithm (Fig. 2).

To enhance the probability of successful use of the screening procedure in clinical practice, we prioritised that the activities were chosen not only due to their psychometric properties but also due to their applicability in clinical practice. Further, to account for the health professionals concerns in terms of patients in delirium or with dementia, the 4QT was chosen as it allows for the relatives to answer the four questions.

The implementation strategy comprises a set of activities targeting health professionals and the organisational level. The activities are divided into activities necessary for adequate delivery of the dysphagia screening procedure (e-learning, teaching and training sessions including a video of the screening procedure, and peer-to-peer training), and supporting the delivery of the screening procedure (electronic detailed description of the procedure and in short as pocket card, a flowchart of the procedure, set-up of the electronic health record, dysphagia ambassadors, evaluation meetings and management support).

**Table 5:** Detailed description of the dysphagia screening intervention comprising screening procedure and implementation strategy

**1. Intervention name**	Dysphagia screening intervention consisting of a dysphagia screening procedure and an implementation strategy.
**2. Why**	In hospitalised geriatric patients, the prevalence of dysphagia ranges from 23 to 82%, leading to potential complications such as malnutrition, dehydration, aspiration, and pneumonia. These complications may result in reduced daily functioning, frailty, extended hospital stays, readmissions, and even mortality. Diagnosing dysphagia in geriatric patients can be challenging due to their multiple health issues and polypharmacy leading to lack of continuity in patient pathways and unnecessary hospitalisations. Timely diagnosis and treatment of dysphagia are crucial and implementation of systematic screening for dysphagia may optimise patient pathways for geriatric patients. The dysphagia screening intervention aims to address this need, aligning with clinical guidelines and political initiatives focused on improving geriatric patient pathways and reducing preventable hospitalisations.
**3. What (materials**)	*The dysphagia screening procedure* • A cup with 30 ml of water • Thickener • A computer for documentation *The implementation strategy* Activities necessary for adequate delivery of the dysphagia screening procedure: • E-learning about dysphagia • Power point presentation, video of the screening procedure (with a QR code), and a manual describing the content used in teaching and training session Activities supporting delivery of the dysphagia screening procedure: • Detailed description of the dysphagia screening procedure in Central Denmark Region’s electronic platform • Pocket card with short description of the dysphagia screening procedure • Flowchart illustrating the screening procedure (Fig. 2) • Set-up of the electronic health record • Video of the documentation in the electronic health record (with a QR code).,
**4. What (procedures)**	*The dysphagia screening procedure* The patient is in upright seated position in bed, on the bedside, or in an armchair. Register if he/she is known to have dysphagia and whether he/she is alert of what is happening. 1. The 4 Questionnaire Test (4QT) [26] consists of four questions about the patient’s habitual ability to eat and drink. If the patient is unable to answer, relatives or caregivers can do so. 2. A 30 ml water swallowing test [27] assesses the patient’s ability to swallow, whether the patient coughs, clears their throat or presence of wet hoarseness. If symptoms of dysphagia are present: 3. Action algorithm (if earlier diagnosed with dysphagia or if the screening procedure indicates dysphagia) with referral for further assessment and treatment by dysphagia specialists (e.g. occupational therapists, speech-language pathologists), and in the meantime nutritional management including considerations of nil per os (intravenous fluids/nasogastric tube). The result is documented in the electronic health record. If dysphagia persists when discharge, this will be included in the discharge documents. *The implementation strategy* Activities necessary for adequate delivery of the dysphagia screening procedure: E-learning includes: - Knowledge about dysphagia - Advice on position during a meal - Modified texture of food and drinks. - Importance of interdisciplinary collaboration regarding dysphagia Teaching and training session includes: - Knowledge about dysphagia, complications and consequences - Importance of multidisciplinary communication and collaboration - Presentation of the dysphagia screening procedure (video) and the flowchart - Practical test of the dysphagia screening procedure and documentation in the electronic health record - Take home message with the three most important learning according to the participants The teaching and training session is described in more details in a manual. For those not participating in the teaching and training session (e.g., temporary workers, being sick or on leave) and in the long term, peer-to-peer training will be provided. Activities supporting delivery of the dysphagia screening procedure: Two dysphagia ambassadors support the delivery of the dysphagia screening procedure in clinical practice. In addition, an occupational therapist is present for support at the medical inpatient ward for the first two weeks. The health professionals are encouraged to write down questions, considerations, praise and criticism. These notes and additional comments are discussed on evaluation meetings.
**5. Who (provided)**	*The dysphagia screening procedure* Provided by multidisciplinary health professionals (nursing staff, occupational therapists or physiotherapists). *The implementation strategy* The teaching and training session is provided by specialists within learning and knowledge sharing, and dysphagia. Participants in the evaluation meetings are the two ambassadors, additional health professionals, a management representative, and if needed, a representative from the development team.
**6. How**	*The dysphagia screening procedure* Delivered individually face-to-face with the patient. If necessary, relatives or caregivers are involved either face-to-face or by telephone to complete the 4QT. *The implementation strategy* The teaching and training session is face-to-face in groups of 10–12 multidisciplinary health professionals. The evaluation meetings are face-to-face.
**7. Where**	Medical inpatient wards
**8. When and How much**	*The dysphagia screening procedure* Conducted within the first 24 h after admission, preferably before fluids and meals are offered. Procedure duration is approximately 5–15 min. *The implementation strategy* Activities necessary for adequate delivery of the dysphagia screening procedure: The 15-minute e-learning is accessible any time, but health professionals are encouraged to conduct it before the teaching and training session, which lasts 1.5 h and is delivered once. Activities supporting the delivery of the dysphagia screening procedure: The 15-minute evaluation meetings are conducted weekly during the first month and afterwards once a month. Once a month, a representative from the development team attends the meeting with an extended timeframe of up to 1 h.
**9. Tailoring**	*The dysphagia screening procedure* The same screening procedure is used for all patients, but variations may occur to meet the needs of the individual patient e.g., patients may not be able to complete the 4QT and/or participate in the 30 ml water swallowing test due to wakefulness, cognition ect. *The implementation strategy* The same teaching and training session is delivered to all health professionals.

### Action 11 End the Development Phase

The development process ended in October 2023. In adherence with the British Medical Research Council framework for development and evaluation of complex interventions [20], we are now planning a feasibility study. Provided the dysphagia screening intervention meets the progression criteria, it will be further refined, and evaluated in a future large-scale study including diverse target populations and several hospital inpatient wards.

## Discussion

We applied a combined a theory-, evidence- and implementation-based approach in combination with stakeholder involvement in the development of a dysphagia screening intervention for geriatric patients admitted to a medical inpatient ward.

The dysphagia screening intervention consists of a screening procedure targeting the patient level, and an implementation strategy targeting health professionals and the organisational level. The screening procedure comprises completion of the 4QT [26], a 30 ml water swallowing test [27], and an action algorithm. The implementation strategy consists of activities necessary for adequate delivery of the dysphagia screening procedure and activities supporting the delivery of the screening procedure. A dysphagia screening intervention combining these activities has not been developed nor feasibility tested or evaluated before.

The dysphagia screening intervention was developed in the context of a medical inpatient ward. It is well-known that contexts can change rapidly during a development process [35]. Despite deliberate choices during the development process, this screening procedure is generic, quick, and simple, and developed with the potential to be generalisable to other target populations and settings. However, a limitation of this screening procedure is that it does not identify patients at risk for silent aspiration; however, to the authors’ knowledge, no existing screening tools currently address this.

Complex interventions are common in healthcare [20]. The dysphagia screening intervention is a complex intervention, consisting of several activities and interacting with the context in which it is implemented [20]. The dynamic, iterative, creative, and open to change development process was guided by The British Medical Research Council framework for developing and evaluating complex interventions [20], and additional frameworks and guiding principles [18, 19, 21, 35], which we believe have been mutually supportive in providing a robust backdrop for the development process. The INDEX guidance supported structuring of the result Sect. [18]. However, literature is scare on similar use of the INDEX guidance [36]. Published reports on development processes make it possible to assess the quality of the process, and there is an association between the development process and the subsequent success of the intervention [18].

To accommodate the complex context in which the screening intervention will be implemented, we prioritised involvement of several groups of stakeholders, who provided unique knowledge and insight into the geographical, epidemiological, socio-cultural, and ethical context domains relevant for development of the dysphagia screening intervention. Engagement of stakeholders can take place at five different levels ranging from information, consultation, involvement, and collaboration to support [37]. The chosen level of stakeholder engagement for our purpose was in the middle of the continuum, as we involved stakeholders directly in the work to ensure that their concerns and aspirations were consistently understood and considered [37]. The dual process of creating a shared understanding, and making relevant and transparent decisions collectively, requires huge investments in e.g., time, finances, and planning, not only in the development team, but also among stakeholders. We aimed for a level of stakeholder engagement balancing investments and expected gains.

The target population, the geriatric patient admitted to a medical inpatient ward, was not represented as a stakeholder group in the development process. This choice is debatable as the perspectives and experiences of geriatric patients are highly important as they have the knowledge of and insight into the entire patient pathway. Initially, we focused on the health professionals as we believed that their potential lack of knowledge about dysphagia, combined with our lack of knowledge about contextual domains was the most distinct barrier to the success of the dysphagia screening intervention. The valuable perspectives of the target population must not be neglected, and their experience of the screening procedure will thus be part of a future feasibility study.

Due to the iterative nature of the development process, the final screening procedure differed from what was initially planned. From the beginning, the ambition was to develop a screening procedure applicable to geriatric patients in both medical inpatient wards and outpatient clinics. When deciding which diagnostic subgroups to include, some were identified as having a higher risk of dysphagia than others e.g., patients with dementia [38], patients with head and neck cancer [39], and patients with chronic obstructive pulmonary disease [40]. However, due to the high prevalence of dysphagia identified in hospitalised geriatric patients in general [2], and to make the implementation easier, it was decided to apply the screening procedure to all geriatric patients despite diagnosis. In terms of the setting, stakeholder involvement, primary data collection, and understanding of the context revealed that the organisation and procedures varied significantly at medical inpatient wards and outpatient clinics, respectively. Therefore, we decided to focus only on the medical inpatient wards.

Some of the implications for future research relate to how many of the potential patients are screened, how many will end up having dysphagia following screening and following assessment by an occupational therapist (reach), and how many of the activities were delivered during the dysphagia screening procedure. In addition, did the screening intervention undergo changes (adaption), and was it delivered as intended (fidelity). It is also important to assess acceptability and potential unintended consequences e.g., enhanced time consumption as experienced by the health professionals, and increased worries or false sense of security as experienced by the geriatric patient. These uncertainties will be addressed in an imminent feasibility study.

In the longer term, there is a demand for assessing the psychometric properties of the screening procedure, and for estimating the duration of admission, readmission, and death in geriatric patients with dysphagia. Finally, a qualitative evaluation will be able to explore what works, for whom, in what circumstances and why in relation to the screening procedure. The need for further research investigating the feasibility and effectiveness of systematic dysphagia screening within this population has been suggested [41].

The introduction of the screening procedure will have some implications for clinical practice. In the medical inpatient ward, it will require time to manage the screening procedure and the related actions. We expect that this will result in a more coherent pathway with fewer days in hospital and fewer readmissions. A positive result of the screening procedure and the recommended actions will be a part of the discharge documents, which will contribute to improve the patient pathway across sectors and thereby the continuity between the secondary (hospital) and primary care settings (general practitioner, municipality health care centre, nursing home etc.). If the screening intervention is proven to be feasible and effective in hospital settings, we believe that it has the potential to be adapted to community-dwelling elderly receiving home nursing care and elderly living in nursing homes, where the prevalence of dysphagia is high [2]. To enhance the quality and probability of success when adapting interventions to new contexts, informed guidance for adapting and transferring interventions to new contexts should be followed [28].

Concerning public health implications awareness has been raised by both politicians and geriatric patients on the complexity of delivering continuous and coherent patient pathways, and on reduction in the number of preventable hospitalisations [17, 42]. Considering the prevalence, complications and consequences, identification and management of dysphagia appears to be essential from a public health perspective, and the introduction of the dysphagia screening intervention may be a step in the optimisation of the geriatric patient pathways.

The systematic development process represents the culmination of many decisions, resulting in a dysphagia screening intervention consisting of a screening procedure targeting the patient level, and an implementation strategy targeting health professionals and the organisational level. The dysphagia screening procedure is now ready for feasibility testing, promising improved health and healthcare services for hospitalised geriatric patients.

## Data Availability

The data supporting the findings of this study are available from the corresponding author upon reasonable request.
